# An MRI-based radiomics nomogram in predicting histologic grade of non-muscle-invasive bladder cancer

**DOI:** 10.3389/fonc.2023.1025972

**Published:** 2023-03-16

**Authors:** Longchao Li, Jing Zhang, Xia Zhe, Hongzhi Chang, Min Tang, Xiaoyan Lei, Li Zhang, Xiaoling Zhang

**Affiliations:** Department of MRI, Shaanxi Provincial People’s Hospital, Xi’an, Shaanxi, China

**Keywords:** radiomics, nomogram, non-muscle-invasive bladder cancer, grade, MRI

## Abstract

**Background:**

Non-muscle-invasive bladder cancer (NMIBC) is categorized into high and low grades with different clinical treatments and prognoses. Thus, accurate preoperative evaluation of the histologic NMIBC grade through imaging techniques is essential.

**Objectives:**

To develop and validate an MRI-based radiomics nomogram for individualized prediction of NMIBC grading.

**Methods:**

The study included 169 consecutive patients with NMIBC (training cohort: n = 118, validation cohort: n = 51). A total of 3148 radiomic features were extracted, and one-way analysis of variance and least absolute shrinkage and selection operator were used to select features for building the radiomics score(Rad-score). Three models to predict NMIBC grading were developed using logistic regression analysis: a clinical model, a radiomics model and a radiomics–clinical combined nomogram model. The discrimination and calibration power and clinical applicability of the models were evaluated. The diagnostic performance of each model was compared by determining the area under the curve (AUC) in receiver operating characteristic (ROC) curve analysis.

**Results:**

A total of 24 features were used to build the Rad-score. A clinical model, a radiomics model, and a radiomics–clinical nomogram model that incorporated the Rad-score, age, and number of tumors were constructed. The radiomics model and nomogram showed AUCs of 0.910 and 0.931 in the validation set, which outperformed the clinical model (0.745). The decision curve analysis also showed that the radiomics model and combined nomogram model yielded higher net benefits than the clinical model.

**Conclusion:**

A radiomics–clinical combined nomogram model has the potential to be used as a non-invasive tool for the differentiating low-from high-grade NMIBCs.

## Introduction

Bladder cancer (BCa) is one of the most common malignant genitourinary tumors and is associated with the highest morbidity and mortality rates. BCa is classified into muscle-invasive bladder cancer (MIBC) or non-muscle-invasive bladder cancer (NMIBC) (1, 2).

NMIBC shows the biological characteristics of multicentric growth and occurs in approximately 75% of BCa cases with a high recurrence rate. Pathological grade is the main independent risk factor most associated with the recurrence of NMIBC (3, 4). Moreover, the prognosis and treatment strategies differ between high and low-grade NMIBCs. The standard treatment for low-grade NMIBC is the transurethral resection of the bladder cancer (TURBT), whereas those with high-grade frequently require more intensive treatment such as radical cystectomy, systemic chemotherapy, and radiation therapy (5–10). On the other hand, high-grade BCa have a higher risk of invading the muscularis propria of the bladder wall and developing into metastatic diseases, which are associated with a poor survival rate (11, 12).

Thus, an accurate preoperative evaluation of the histologic grade of NMIBC is of great clinical significance and prediction of recurrence. Currently, cystoscopic biopsy remains the standard for assessing NMIBC grade. However, this examination is invasive and expensive. Moreover, biopsy results are not always representative of the entire tumor, which may lead to misdiagnoses (13, 14).

Notably, researchers have found that age, tumor size, number of tumors, and sex are risk factors for bladder cancer grading (15–17). Moreover, previous studies reported that MRI shows good performance in diagnosis and prediction of NMIBC grade, especially for ADC values (18–20). Furthermore, radiomics can be used to quantify the morphological features and internal heterogeneity of the lesions, and obtain information that cannot be determined by subjective evaluations for disease diagnosis and evaluation (21).

Recent studies showed that radiomics based on MRI can be used as an accurate and noninvasive imaging tool for preoperative prediction of the pathological grade of BCa (22, 23). However, none of the prior studies arrived at a consensus regarding the performance of radiomics in distinguishing low- and high-grade NMIBC. On the basis of these observations, we aim to (1) develop and validate a nomogram combing radiomics based on MRI and important clinical factors for preoperative prediction of the histological grade of NMIBC and (2) compare the diagnostic performance of a clinical model, a radiomics model, and a radiomics–clinical nomogram model.

## Materials and methods

This retrospective study was approved by the institutional Ethics Review Board, which waived the requirement for obtaining written informed consent, and was performed according to the TRIPOD reporting checklist (available at http://dx.doi.org/10.21037/tau-21-49).

### Patients

A total of 202 consecutive patients with pathologically confirmed NMIBC who underwent multiparametric MRI examinations from September 2017 to December 2021 were enrolled in our study. Inclusion criteria were as follows: (1)postoperative pathologically confirmed NMIBC for the first time; and (2) 3.0 T MRI scans performed<1 month before surgery, with the sequence including T2WI and DWI scans with b values of 0 and 1000mm^2^/s.

**Figure 1:**
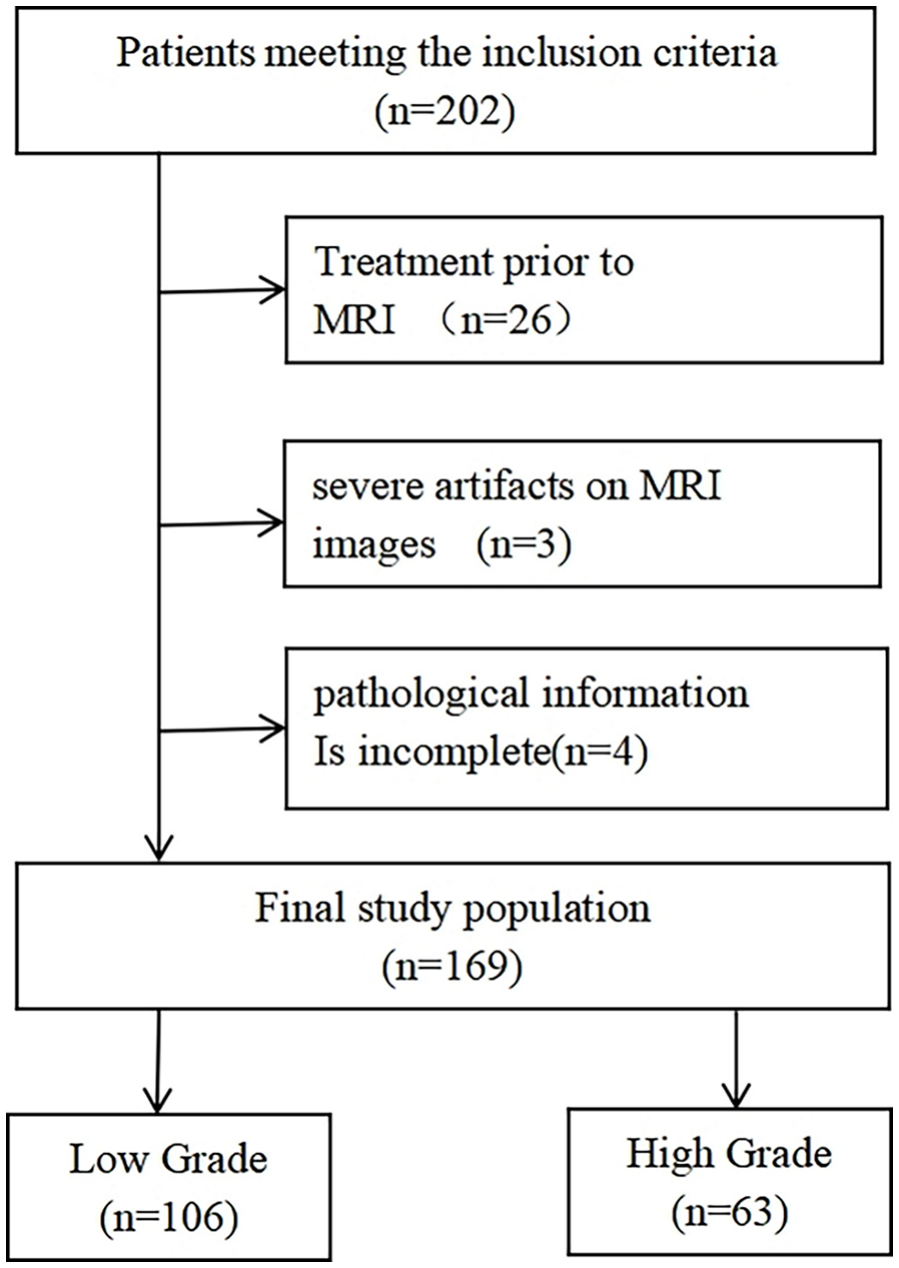
Flow diagram of patient recruitment.

The exclusion criteria were as follows: (1) a history of any treatments, including chemotherapy, radiotherapy, TURBT, or BCG, before the pelvic MRI; (2) presence of severe artifacts that could make segmentation of cancer difficult on MRI scans; and (3) incomplete pathological information. Finally, 169 patients were found to be eligible in this study and divided into low- and high-grade groups (low-grade, 106 patients; high-grade, 63 patients) according to their pathological results. **Figure 1** shows the flow diagram of patient recruitment.

### MRI acquisition

All images were acquired using a 3.0TMR scanners(Ingenia and Ingenia CX, Philips Healthcare, and the Netherlands) with a 16-channel body phased array coil. A standard MRI protocol included sagittal T2WI, axial T2WI, and DWI.Turbo-spin-echo non-fat-suppressed T2WI (TR/TE, 2516-3626/100) was performed with a slice thickness of 4 mm. DWI was performed with a breathing-free spin-echo planar imaging sequence in axial view including a high b-value (1000 s/mm^2^) and a slice thickness of 4 mm. Apparent diffusion coefficient (ADC) maps were automatically reconstructed on a designated workstation. The detailed parameters of the MRI examinations are shown in **Table S1(Supplemental Material)**.

### 3D region of interest (ROI) delineation

**Figure 2:**
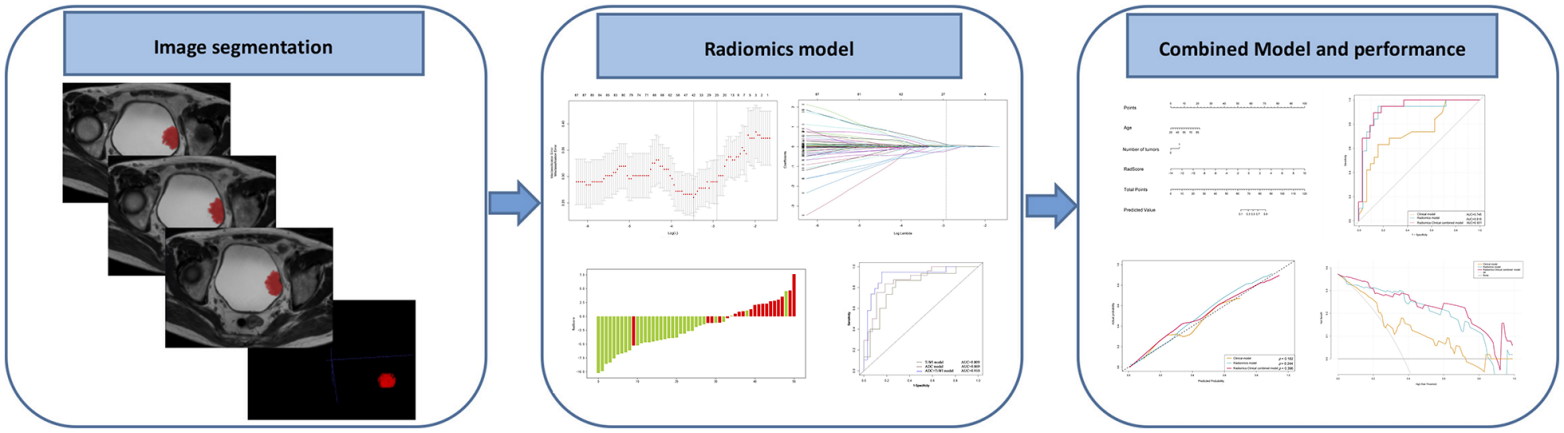
Radiomics workflow and study flowchart.

Two radiologists with no prior knowledge of the histopathological results (L.L.C. and Z.L., with 6and 5 years of experience in BCa imaging, respectively) manually delineated the ROIs slice-by-slice along the tumor contour on axial T2WI maps using ITK-SNAP software. Then, the divergence of their delineation results was carefully corrected in consensus. The ROIs obtained from T2WI images were mapped on the ADC maps to obtain the corresponding tumor region. **Figure 2** shows an example of a lesion ROI delineated on T2WI mapping for enrolled patients.

### Imaging feature extraction and selection

This study used an open-source Python package (Pyradiomics 2.0.1) for feature extraction. In this study, the radiomics features belonged to five categories: 1) first-order statistics; 2) shape and size; 3) texture; 4) wavelet filter; and 5) Laplacian of Gaussian filter features.

A total of 3148 features were extracted from each T2WI and ADC map. The intraobserver and interobserver repeatability of lesion segmentation was evaluated to select stable and reproducible features. Only the features with inter-and intra-class correlation coefficient (ICC) > 0.75 were included in the following analysis.

To identify the features showing the greatest differences in the high- and low-grade NMIBC groups, one-way analysis of variance (ANOVA) was performed to select the optimal features for predicting grade (24, 25). Second, the least absolute shrinkage and selection operator (LASSO) regression method was applied to select the most discriminating radiomics features before classification.

### Development of the clinical model

The clinical factors, including age, sex, tumor size and number of tumors, were assessed by univariate logistic regression. To build the clinical model, a multivariate logistic regression analysis was conducted to assess the features identified as statistically significant in univariate logistic regression analysis.

### Development of the radiomics model

The patients were subsequently randomly divided into training and validation sets at a ratio of 7:3. On the basis of the characteristics selected in the above procedures, a logistic regression algorithm was used to build the prediction model for NMIBC grading with the training set and the independent validation cohort based on T2WI and ADC mapping. This model generated an internal score called decision value that was used as the Rad-score.

### Development of the radiomics–clinical nomogram

The Rad-score and the potential clinical factors, including age, sex, tumor number, and tumor size were introduced into univariate and multivariate regression to select independent predictors. A nomogram was constructed with these independent significant risk factors, which is a visualized and individual tool to predict the probability of NMIBC grade in the training cohort.

### Validation of the three predictive models

The area under the curve (AUC) of the receiver operating characteristic (ROC) curve, accuracy, sensitivity, and specificity were calculated to determine the discrimination performance for NMIBC grading. For all three models, calibration curves were derived from the regression analysis to assess the predicted and the actual outcomes.

### Clinical usefulness

The decision curve analysis was used to investigate the clinical usefulness of the three models by quantifying the net benefits at different threshold probabilities in both the training and the validation datasets (26).

### Statistical analysis

Statistical Package for Social Science (SPSS) 22.0 and MedCalc version 15.2.2, R software version 4.1.2, Pyradiomics version 2.0.1. were used for statistical analysis. The chi-square test and Mann–Whitney–Wilcoxon U test were used to compare group differences. The univariate and multivariate regression models were performed to determine the independent predictors from the clinical factors and Rad-score to differentiate high- and low-grade NMIBC. The odds ratios and 95% confidence intervals were calculated. The diagnostic abilities of the three predictive models were finally compared using the DeLong test. *P* values less than 0.05 were considered statistically significant.

## Results

### Clinical characteristics of the patients

On the basis of computer-generated random numbers in a 7:3 ratio, the 118 patients were grouped into the training cohort, and the remaining 51 patients were included in the validation cohort. The two groups showed no significant difference in clinical characteristics (*P*>0.05). **Table 1** shows the baseline demographic and clinical characteristics of patients in the training and validation cohorts.

**Table 1:** The baseline demographics and clinical characteristics of patients in the training and validation cohorts.

Characteristics	Training cohort (n= 118)	Validation cohort (n =51)	*P* value
Gender, No. (%)			
Male	96 (81.4)	40 (78.4)	0.660^a^
Female	22 (18.6)	11 (21.6)	
Age, median (range), years	65 (27, 89)	63 (29, 87)	0.670^b^
<65 years, No. (%)	57 (48.3)	27 (52.9)	
≥65 years, No. (%)	61 (51.7)	24 (47.1)	
Grade, No. (%)			
High grade	44 (37.3)	19 (37.3)	0.997^a^
Low grade	74 (62.7)	32 (62.7)	
Number of tumors^c^, No. (%)			
Single	86 (72.9)	38 (74.5)	0.826^a^
Multiple	32 (27.1)	11 (21.6)	
Tumor size^c^, median (range), cm	1.7 (0.6, 4.5)	1.5 (0.5, 4.2)	0.459^b^

a Statistical analysis performed using chi-square test.

b Statistical analysis performed using Mann–Whitney U test.

c MRI-determined information.

### Development and performance of the clinical model

The univariate and multivariate logistic regression analysis showed that age and number of tumors were independent predictors of high-grade NMIBC. The logistic regression classifier was established based on the selected clinical features, including age and number of tumors. The AUC, sensitivity, and specificity of the clinical model were 0.723, 0.795, and 0.594 in the training set and 0.745, 0.632, and 0.844 in the validation set, respectively.

### Feature selection and radiomics signature construction

The interobserver and intraobserver ICCs of the selected features ranged from 0.776 to 0.998. On the basis of the threshold ICC value of >0.75, all the 3148 features were extracted from T2WI and ADC images. After feature selection, 24 optimal radiomics features were selected using the ANOVA and LASSO methods, and these features were defined as the radiomics signature (**Table S2 in the Supplemental Material**).

**Figure 3:**
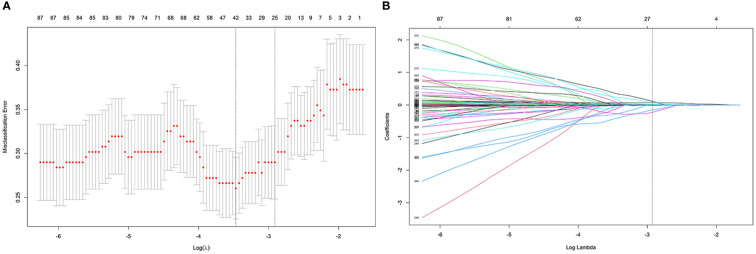
The process of feature selection using the LASSO algorithm. **(A)** Selection of the tuning parameter λ in the LASSO classififier via 10-fold cross-validation based on minimum criteria. **(B)** LASSO coefficient profiles of the 24 radiomics features.

**Figure 4:**
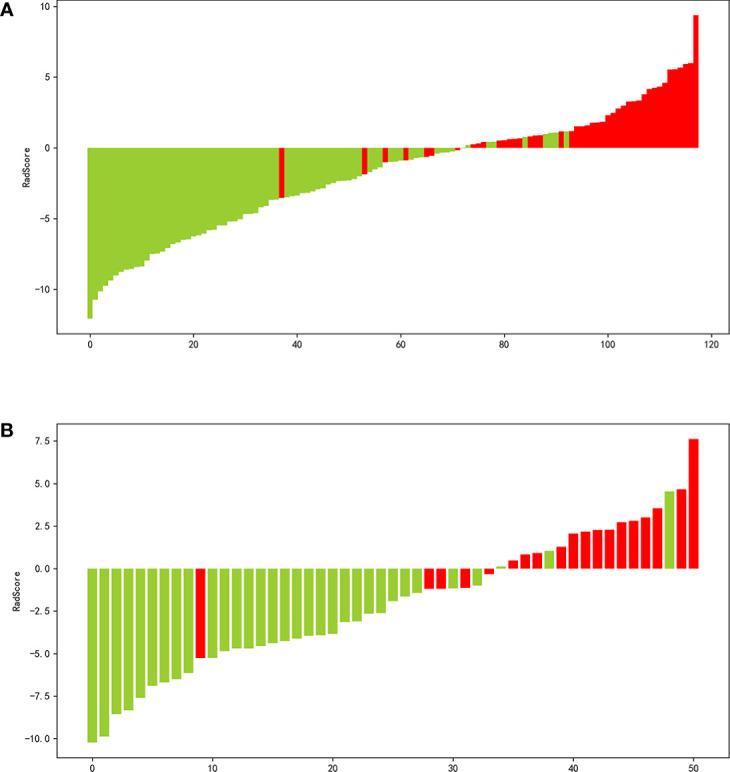
Waterfall plot of the distribution of radiomics score and pathological grade of individual patients in the in the training **(A)** and validation **(B)** sets.

The process employed in the LASSO binary logistic regression model is shown in **Figure 3**. The distributions of the Rad-score and NMIBC status in the training and validation cohorts are shown in **Figure 4**.

### Validation of the radiomics model

**Figure 5:**
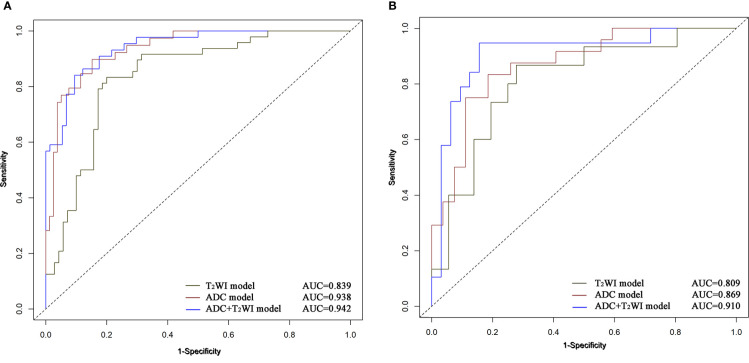
ROC curves of Logistic Regression classifiers using 3 radiomics models in the training **(A)** and validation **(B)** cohors.

The ADC and T2WI imaging models yielded AUC values of 0.938 and 0.839, respectively, for the training cohort and 0.869 and 0.809, respectively, for the validation cohort. The radiomics signature of the ADC and T2WI imaging fusion model showed higher predictive efficiency with AUC values of 0.942 in the training set and 0.910 in the validation set in comparison with the models based on single MR images. The ROC curves are shown in **Figure 5**.

### Development and performance of the nomogram

**Figure 6:**
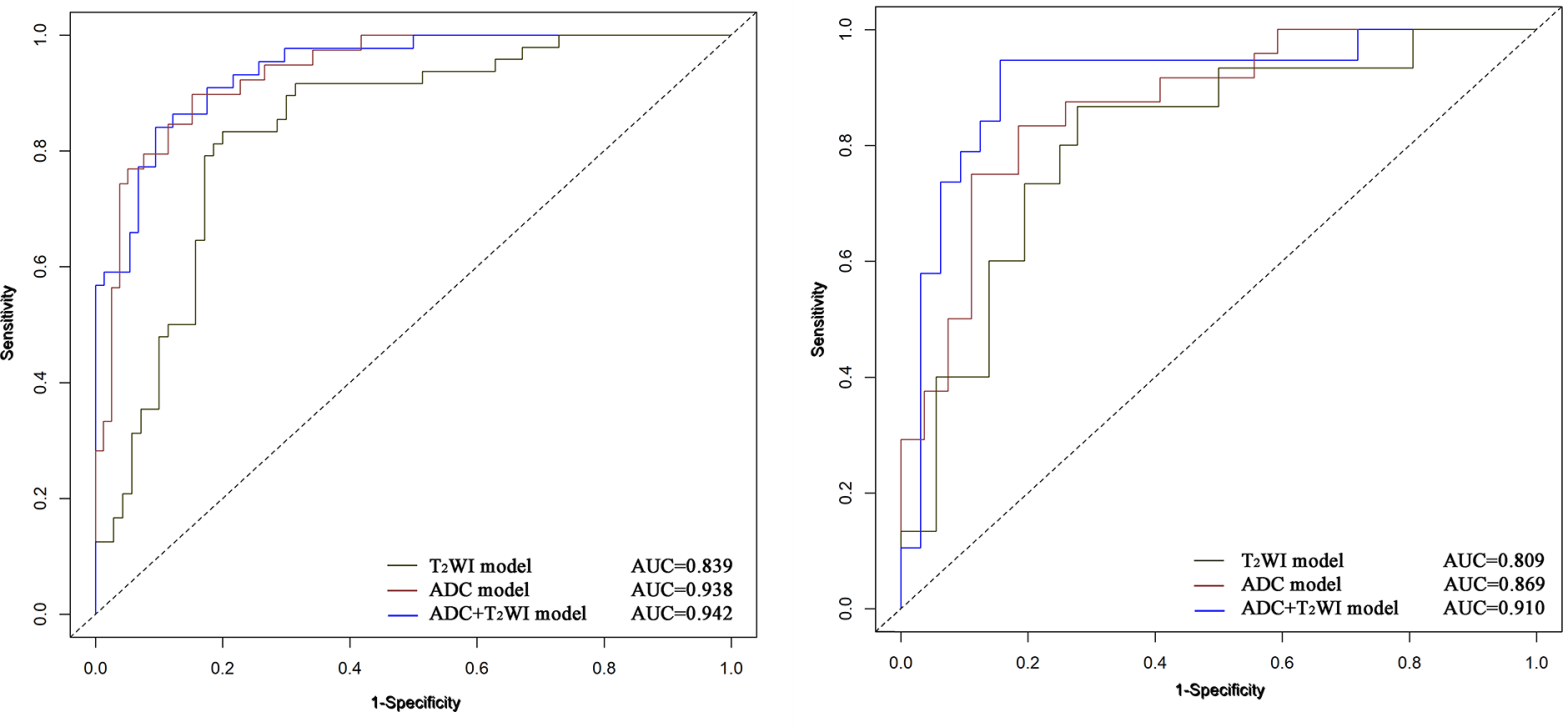
The nomogram integrating the radiomics score and independent risk factors was constructed to predict the pathological grade of NMIBC.

**Figure 7:**
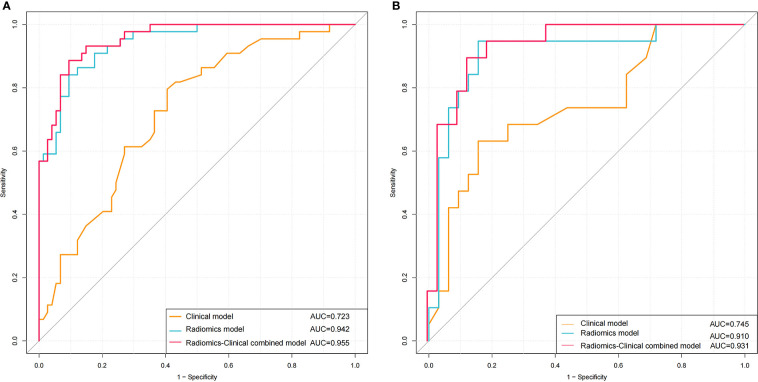
ROC curves of using Clinical model, Radiomics model and Radiomics-Clinical combined model to predicting the pathological grade of NMIBC in the training **(A)** and validation **(B)** cohorts.

The univariate and multivariate logistic regression analysis showed that age, number of tumors, and Rad-score were independent predictors of high-grade NMIBC (**Table 2**). A radiomics–clinical nomogram for NMIBC grading was developed by using the independent risk factors mentioned above, as shown in **Figure 6**. Theradiomics–clinical nomogram showed good predictive ability with high AUC values of 0.955 in the training set and 0.931 in the validation set, which were slightly better than the radiomics model. The AUC, accuracy, sensitivity, and specificity of the three models are listed in **Table 3**. A comparison of the ROC curves of these three models is shown in **Figure 7**.

**Table 2:** Risk factors for high grade in non-muscle-invasive bladder cancer.

Variables	Univariate analysis		Multivariate analysis	
	OR(95% CI)	*P* value	OR(95% CI)	*P* value
Gender	0.655 (0.303-1.414)	0.281	–	–
Age	1.064 (1.304-1.095)	<0.001	1.068 (1.017-1.121)	0.009
Tumor size	1.415 (0.980-2.043)	0.064	–	–
Number of tumors	2.491 (1.240-5.004)	0.01	3.530 (1.161-10.721)	0.026
Rad-Score	2.269 (1.744-2.952)	<0.001	2.274 (1.720-3.005)	<0.001

**Table 3:** Performance of clinical and radiomic models.

Model		AUC	Sensitivity	Specificity	Accuracy	*P* value
Training cohort						
Clinical model		0.723	0.795	0.594	0.669	
Radiomics model	T_2_WI	0.839	0.833	0.800	0.814	
	ADC	0.938	0.897	0.848	0.864	
	ADC+T_2_WI	0.942	0.841	0.905	0.881	<0.001^d^
Combined model		0.955	0.886	0.905	0.898	0.214^e^
Validation cohort						
Clinical model		0.745	0.632	0.844	0.765	
Radiomics model	T_2_WI	0.809	0.867	0.722	0.765	
	ADC	0.869	0.833	0.815	0.824	
	ADC+T_2_WI	0.910	0.947	0.844	0.882	0.026^d^
Combined model		0.931	0.895	0.875	0.882	0.277^e^

d Comparison of ROC curve performance between clinical model and radiomics model using DeLong test.

e Comparison of ROC curve performance between radiomics model and radiomics-Clinical combined model using DeLong test.

**Figure 8:**
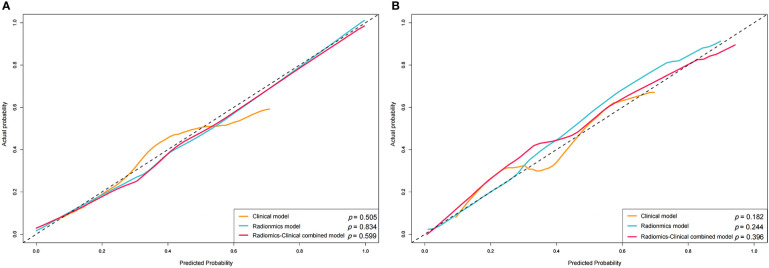
Calibration curve of the nomogram in the training **(A)** and validation **(B)** sets.

The calibration curve showed good agreement between predictions and observations both in the training set and validation set (**Figure 8**). The Hosmer-Lemeshow test yielded non-significant *P* values in both cohorts (*P* = 0.599 and 0.396), which suggested good calibration.

### Clinical usefulness

**Figure 9:**
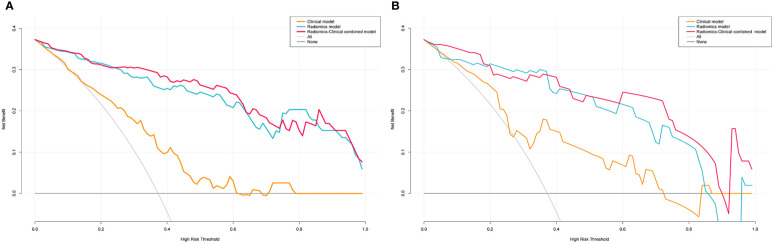
DCA for Clinical, Radiomics and Radiomics-Clinical nomogram in the training **(A)** and validation **(B)** sets.

The decision curves for the clinical, radiomics, and combined nomogram models are presented in **Figure 9**. The decision curves showed that using the radiomics or combined nomogram models to predict high-grade NMIBC was more beneficial than using the clinical model.

## Discussion

Noninvasively accurate predicting histologic grade of NIMBC is of great significance because high-and low-grade cancers have different clinical treatments and prognoses. In our study, we developed and validated three models for preoperative assessment of the pathological grade of NMIBC. The radiomics model and the nomogram model combining the optimal radiomics signature and clinical risk factors, including age and number of tumors, showed better diagnostic ability for NMIBC grading than the clinical model. Thus, inclusion of the Rad-score in the clinical model improved the diagnostic efficiency of clinical factors, and achieved novel calibration and good clinical net benefit, indicating its usefulness for NMIBC grading.

On the other hand, this study also revealed that combined T2WI and ADC mapping showed higher performance than assessments based on a single imaging modality (AUCs of 0.910 vs. 0.869 and 0.809, respectively). The grading performance of ADC mapping was better than that of the T2WI model (AUCs of 0.869 and 0.809, respectively). This finding can be attributed to the ability of ADC to describe intratumoral characteristics and heterogeneity and radiomics based on ADC maps can describe the contrast, complexity and heterogeneity of local intensity patterns, and potentially have more discriminative power cancer grading. Considering that NMIBC of different pathological grades would have different diffusion patterns of water molecules, different image phenotypes may be better presented in ADC maps (27, 28). While T2WI only provides the morphological features of NMIBC on the basis of signal intensity (15, 29). In addition, ADC- and T2WI-based radiomics features have been commonly used to develop radiomics models because they can be used without contrast agents for several tumors and can facilitate clinical decision-making.

Several studies have investigated the grade of BCa, but we were unable to find studies that examined grading of NMIBC (22, 23). Wang et al. reported an MRI-based radiomics approach that showed good discrimination for high-grade versus low-grade tumors, with an AUC of 0.9233 in the training cohort and 0.9276 in the validation cohort (22). Zheng et al. reported that a radiomics–clinical nomogram showed good discrimination both in the training set (AUC: 0.956) and validation set (AUC: 0.958) (23). Zhang et al. reported that a combination of textural features from DWI and ADC maps achieved the best performance in BCa grading, with an AUC of 0.861, a sensitivity of 0.784, and a specificity of 0.871 (15).

Similar to the previous study, our results also suggest that a radiomics signature based on MRI can be used to make noninvasive, preoperative predictions of the tumor grade of BCa. However, about 75% of patients are limited to mucosa, submucosa and carcinoma in situ, which is belong to NMIBCs, with biological characteristics of high recurrence and progression risk (30). Preoperative prediction of high-grade NMIBC is thus of great significance for treatment planning and prognosis evaluation (9, 10).

To our knowledge, our study is the first to develop a radiomics model for NMIBC grading. Our findings revealed the strengths of radiomics-based approaches in comparison with the clinical model. Clinical risk factors could not reflect the intratumor heterogeneity. In addition, the number of tumors was subjective. Preoperative pathological grading of BCa mainly depended on cystoscopy biopsy, but it was invasive examination, local sampling was easy to underestimate the staging, could not reflect the overall heterogeneity of the tumor, and could not observe the invasion of the tumor outside the bladder wall (14, 31).

In contrast, radiomics could extract most of the objective quantitative features (including shape- and size-based features, first-order features, textural features, and wavelet features) mentioned in the current literature from a 3D region, providing a comprehensive evaluation of intratumor heterogeneity (32).

Since tumors of different pathological grades would have different diffusion patterns of water molecules, differences in image phenotypes may be better presented in ADC maps based on radiomics (29, 33). Currently, a single model or feature may no longer satisfy the development of precision medicine. Only a comprehensive utilization of potentially useful features can further improve the performance of the models (34).

The nomogram is a perfect example of integration of multiple related parameters to predict a specific end point by means of geometry graphs to visualize the results of the prediction model (35, 36). With the nomogram, we could intuitively obtain a patient’s corresponding risk value for high-grade NMIBC on the prediction line at the bottom of the nomogram, which was then be used to guide the urologists’ decision on conducting a second TURBT and intravesical chemotherapy.

The AUC of the combined clinical factors and radiomics nomogram was 0.931 for the validation cohort, indicating that the constructed model showed satisfactory predictive accuracy for high-grade NMIBC. Furthermore, the calibration curve revealed good consistency between the actual and predicted outcomes. In addition, for assessing the viability of the prediction model, we conducted a decision curve analysis. The results showed that using the prediction model to predict the risk of high-grade NMIBC could yield higher net benefit than treating all or no patient in most ranges of the threshold probabilities. Therefore, the combined nomogram prediction model offered advantages in guiding physician decision-making, but it cannot replace the cystoscopy and histological evaluation.

Our study had several limitations. First, this was a retrospective study and conducted at a single center, so potential selection biases may have occurred. Thus, prospective, larger, multicenter studies are required to evaluate the general ability of the proposed nomogram. Second, our study only included T2WI and ADC mapping. The addition of other series, such as dynamic contrast-enhanced or perfusion-weighted images and high b-value images, may provide more information and improve performance (37, 38). Thirdly, VI-RADS is the preferred method for multi-parameter MRI to evaluate the staging of BCa. The grading value of VI-RADS needs be further analyzed in the future. Fourth, our study did not analyze the carcinoma *in situ* separately because the small sample size. In the future, we will expand the sample size for further analysis. In conclusion, our results preliminarily demonstrate that a nomogram based on radiomics features together with the important clinical risk factors can be used to non-invasive assess tumor grade in NMIBC, which may help radiologists and urologists in discriminating between low- and high-grade NMIBCs more precisely.

## Data availability statement

The original contributions presented in the study are included in the article/**Supplementary Material**. Further inquiries can be directed to the corresponding author.

## Ethics statement

This retrospective study was approved by the institutional Ethics Review Board, which waived the requirement for obtaining written informed consent, and was performed according to the TRIPOD reporting checklist (available at http://dx.doi.org/10.21037/tau-21-49).

## Author contributions

Among the authors in the list, LL and LZ have done a lot of work in research design, data collection, paper writing modification, and paper finalization. XZ and JZ not only participated in the data collection and writing of the paper. XLZ made great efforts in the design and drafting of this paper. MT made some contributions to the data analysis and writing of the article. HC and XL have done a lot of work in research design, data analysis, and paper finalization. All authors contributed to the article and approved the submitted version.
